# Human and Bovine Tuberculosis

**Published:** 1904-06-11

**Authors:** 


### Human and Bovine Tuberculosis.

From the time that Professor Koch uttered his famous dictum as to the dissimilarity of human and bovine tuberculosis the tide of opposition to his views has been steadily rising, and the evidence contradicting them has been increasing; until the successive experiments of Professors Crookshanks, Revenel, Delépine, and Hamilton have prepared the way for the conclusions which the Royal Commission now place beyond the reach of doubt. Questions of scientific detail may wait for more complete elucidation hereafter, but the broad fact of the identity of human and bovine tubercle bacilli must be at once accepted as governing both the course of legislation and the nature of domestic precautions against the diffusion and communication of tuberculous disease. We mentioned the main lines of the Commission's interim report in an article last week, but it may be not unprofitable to consider some of the lessons which this document ought to convey.

The sort of blind submission with which a certain school of bacteriologists in this country have been wont to receive as authentic the opinions advanced by Professor Koch is hardly likely, we think, to survive this most recent evidence of the fact that eminent German authorities share to the full in the fallibility of human nature. There can be no reasonable doubt of their skill in certain departments of observation; there can be as little that this skill has sometimes tempted them to speak as if *ex cathedrâ* upon questions as to which they are in no special way qualified to decide. It is one of the intellectual vices of our time to assume that a man who is powerful and sagacious in one direction must of necessity be equally powerful and sagacious in others; and it is only minds of the very highest order which are proof against the temptations to profess omniscience which are thrown in their way by their admirers. During the heyday of the popularity of Mr. Gladstone, for example, the newspapers of his party were not content with extolling him as a statesman and a financier, but they also hung entranced upon his utterances concerning the domestic manufacture of jam. Notwithstanding the complete disappointment of Professor Koch's expectations concerning tuberculin, we were seriously told, a few months ago, that, because he had declared human and bovine tuberculosis to be different diseases, we ought at once to lay aside all the precautions which the previous belief in their identity had rendered manifestly prudent, to relax or to abandon legislation, and to conform our conduct in all respects to the dicta of the oracle —dicta which, after all, were founded upon nothing more than the failure of inoculations which, if we may judge by the evidence now in our possession, must have been inefficiently performed. A very short time after their publication they were repeated in this country with diametrically opposite results, and these results have now been confirmed upon a more extended scale and by an authority not to be gainsaid by declarations "made in Germany." These declarations have, no doubt, done a certain amount of mischief and have placed certain impediments in the way of much-needed legislation and practice. We can only hope that their consequences may be as ephemeral as the errors which furnished their sole foundation.

It is too much, we presume, to expect from a Parliament which is approaching the natural term of its existence, and which is likely to be fully occupied in the improvement of our representative system, that it should take any serious heed of the vast importance of the questions underlying the general feeding of the nation—questions by which the intellectual and physical capacities of the people are likely to be governed, not only in the immediately rising generation, but in many of those which will succeed it. We are urged by statesmen to "think imperially," and to fit ourselves in every way for the position of an imperial race; and it is quite certain that, if we fail to do so, we shall have no lack of rivals ready to dispute our supremacy. In the words of a very homely proverb, children are like young horses; all that they are good for goes in at their mouths; and this is probably even more true in relation to the earliest years of life than it is in relation to those which follow them. Notwithstanding this, we leave the girls of our working classes to grow up without instruction concerning the management and feeding of children, and we allow the common advertiser to poison the minds of the ignorant with falsehoods concerning the nutritive value of the rubbish which brings money into his pockets. Our Medical Officers of Health throughout the country are commencing an endeavour, destined, we hope, to be successful, to obtain proper fixity of tenure for the body to which they belong, and to prevent dismissal from following, as it now does, closely in the wake of duty. If they obtain the conditions sought for—the only ones, be it observed, under which their work can be properly performed—they may reasonably be expected to assist in promoting a general knowledge of the absolute money-value of health to the community, and of the suicidal character of a laxity of legislation which allows that health to be played with or sacrificed for the benefit of a few tradespeople. Diseased meat, diluted milk, innutritious bread, and worthless "patent" foods, are prominent among the agencies by which the powers of the rising generation are being undermined; and it should be one of the most important duties of the Legislature to see that the effects of these agencies are neutralised by the careful administration of wise enactments.

